# Outcomes of fertility preservation treatments in patients with endometrial cancer with different molecular classifications based on an NGS panel

**DOI:** 10.3389/fonc.2023.1282356

**Published:** 2023-11-09

**Authors:** Yan Xu, Mingming Zhao, Li Zhang, Tianyou Wang, Bo Wang, Yu Xue, Zhiying Xu, Wenyu Shao, Xiaojun Chen, Chao Wang

**Affiliations:** Obstetrics and Gynecology Hospital, Fudan University, Shanghai, China

**Keywords:** endometrial cancer, NGS - next generation sequencing, fertility preservation treatment, molecular classifcation, molecular features

## Abstract

**Background:**

The molecular classification of endometrial cancer has previously been shown to be associated with clinical outcomes. However, there are insufficient data to support the routine use of molecular classification for the treatment of patients seeking fertility preservation.

**Methods:**

Here, we retrospectively investigated 90 patients received fertility-sparing treatment. We used a next generation sequencing (NGS) panel to classify these patients into four subtypes. All patients received hormonal therapy combined with hysteroscopy. Therapeutic effects were evaluated by hysteroscopy every three months during the treatment.

**Results:**

Patients with POLE mutations had the highest disease progression rate (50.0%, P=0.013), while the microsatellite instability-high (MSI-H) group had the highest recurrence rate (50.0%, P=0.042). *PIK3CA* mutation (hazard ratio (HR): 0.61; 95% confidence interval (CI): 0.37–0.99; P=0.046), overweight (HR: 0.56; 95% CI: 0.32–0.96; P=0.033) and obesity (HR: 0.44; 95% CI: 0.20–0.95; P=0.036) were associated with a significantly lower cumulative complete response (CR) rate. The combination of gonadotropin-releasing hormone analogues (GnRH-a) and letrozole (HR: 3.43; 95% CI: 1.81–6.52; P< 0.001) was associated with a significantly higher cumulative CR rate. *KRAS* mutation was significantly associated with disease progression (P=0.002). In wild-type TP53 patients, *PTEN* and *PIK3CA* mutations significantly prolonged the duration of treatment to achieve CR (log rank P=0.034; P=0.018).

**Conclusion:**

The implementation of molecular classification for EC patients undergoing fertility-sparing treatment is promising and can facilitate the selection of appropriate medical regimes to achieve better outcomes in patients with EC who require fertility preservation treatment.

## 1 Introduction

The incidence rates of endometrial cancer (EC) have been rising over recent years, with an estimated 65,950 new cases and 12,550 deaths in the U.S.A in 2022 (1). Early-onset endometrial cancer (EOEC), diagnosed in patients under 50 years-of-age, is relatively uncommon, while recent studies have indicated that the incidence of EC is rising continually among young patients, particularly in women under the age of 45 (2, 3). According to the National Cancer Institute, the incidence rates of EC among women aged 20-34 years and 35-44 years were 1.8% and 5.3%, respectively (4). This implied that the proportion of cases managed by fertility-sparing treatment (FST) is increasing when compared to hysterectomy in young patients with early-stage endometrial cancer. Currently, the majority of FST studies related to EC focus mostly on assessing the treatment effects of various therapies and identifying clinical factors that impact FST outcomes (5–7). However, as research advances, studies of EC are transitioning toward a molecular-based approach.

The molecular classification of EC was first proposed by The Cancer Genome Atlas (TCGA) in 2013, which classified EC into four subtypes based on array and sequence technologies: (1) POLE ultramutated, (2) microsatellite instability hypermutated, (3) copy-number low, and (4) copy-number high (8). Subsequently, clinically applicable molecular classification systems were developed based on immunohistochemistry (IHC) or next generation sequencing (NGS) (9–12). NGS has been established to represent an effective method for the molecular classification of EC and shows high concordance with the final hysterectomy specimens when applied to curettage samples (13). According to NGS molecular classification, EC can be divided into four subtypes: (1) *POLE* mutated (*POLE* mut), (2) microsatellite instability hypermutated (MSI-H), 3. *TP53* wild-type (*TP53* wt), and (4) *TP53* abnormal (*TP53* abn). Both National Comprehensive Cancer Network (NCCN) and ESGO-ESMO-ESTRO have included molecular classification as a consideration to guide treatment strategies for EC patients undergoing surgery (14, 15). In addition, molecular classification has been encouraged in the newest ESGO/ESHRE/ESGE guidelines for EC patients receiving FST (15).

There were only a limited number of studies exploring the relationship between molecular classification and FST for patients with EC. However, these studies reported different outcomes. Some studies suggested that different molecular subtypes responded differently to conservative treatment, and that mismatch repair deficiency (MMR-D) may be associated with a longer treatment duration and a higher risk of recurrence than other subtypes (16, 17). However, another study indicated that molecular classification might not exert prognostic significance for EC patients undergoing FST (18). Consequently, there is an urgent need for further clinical research to confirm the significance of molecular classification for patients with EC undergoing FST.

In this single-center retrospective study, we aimed to evaluate the efficacy of FST among different molecular subtypes in patients with EC. Furthermore, we aimed to identify novel molecular prognostic factors for FST by comprehensively analyzing genomic changes in patients with EC by NGS testing.

## 2 Materials and methods

### 2.1 Study population

In this retrospective study, we investigated all EC patients receiving FST in the Obstetrics and Gynecology Hospital of Fudan University between January 2021 and January 2022. These patients include those who were initially diagnosed with EC and those who progressed to EC during the course of treatment. The study was approved by Ethics Committees of Obstetrics and Gynecology Hospital of Fudan University.

The diagnosis of EC was confirmed by at least two experienced gynecological pathologists according to the World Health Organization (WHO) Pathological Classification of EC (2014). Tissue specimens were obtained by dilation and curettage (D&C) with or without hysteroscopy.

The criteria for inclusion were as follows: (1) aged between 18 and 45 years, (2) a strong desire to preserve fertility, (3) histologically proven endometrial endometrioid carcinoma (EEC) upon initial diagnosis or progressed from atypical endometrial hyperplasia (AEH) during FST, (4) disease limited to the endometrium as observed and no suspicious or metastatic lesions by enhanced magnetic resonance imaging (MRI) or transvaginal ultrasound, (5) non-pregnant state, (6) no contraindication for progestin treatment, and (7) molecular classification of an endometrial lesion obtained prior to the initiation of treatment. The criteria for exclusion were as follows: (1) a history of local or systemic hormone treatment for more than one month prior to initial evaluation and treatment in our center, (2) specimens had insufficient DNA quality for NGS, and (3) patients transferred to another hospital during the treatment.

### 2.2 Diagnosis and assessment

General information (including age, weight, height) and serum samples were collected prior to any form of treatment. All serum samples were collected and examined in the laboratory at the Obstetrics and Gynecology Hospital, Fudan University.

Body mass index (BMI) was calculated as weight (kg)/height (m2); a BMI ≥25 kg/m^2^ was considered as overweight while a BMI ≥30 kg/m^2^ was considered as obesity. According to our previous study (19), we considered a patient to be insulin resistant (IR) when the homeostasis model assessment-insulin resistance (HOMA-IR) was ≥2.95.

### 2.3 Treatment and evaluation

Patients who met the inclusion criteria received comprehensive evaluation, and a multidisciplinary team decided the therapeutic regimens for each patient. All patients received one of the following therapies: (1) oral megestrol acetate (MA) at a dose of 160 mg/d; (2) oral medroxyprogesterone acetate (MPA) at a dose of 250 mg/d; (3) levonorgestrel intrauterine system (LNG-IUS) insertion; (4) oral MA at a dose of 160 mg/d combined with LNG-IUS, or (5) gonadotropin-releasing hormone analogues (GnRH-a) at a dose of 3.75 mg/4w (intra-muscular [i.m.]) combined with oral letrozole at a dose of 2.5 mg/d. Some patients also received oral metformin at a dose of 1500 mg/d or rosuvastatin at a dose of 10 mg/d, depending on their medical complications.

Complete hysteroscopic evaluation was performed every three months during medical treatment to evaluate the efficacy of FST. Endometrial lesions were removed under hysteroscopy, and endometrium biopsy was performed if no obvious lesion was found. The cut-off points for analysis were extended to the 16^th^ and 32^nd^ weeks to account for slight variations in the timing of hysteroscopic evaluations.

The response to hormone treatment was assessed histologically using specimens obtained during each hysteroscopic evaluation. Complete response (CR) was defined as the absence of hyperplasia or cancer. Partial response (PR) was defined as pathological improvement. No response (NR) was defined as the persistence of EC. Progressive disease (PD) was defined as any appearance of disease with a higher degree, such as a higher histological grade, deep myometrial invasion, and/or extrauterine lesions. Recurrence was defined as atypical hyperplasia or carcinoma developing after CR was achieved. Time to CR was measured from the time point at which treatment was initiated to the time point at which CR was diagnosed pathologically by hysteroscopy.

Patients ceased or changed FST if unacceptable side effects occurred any time. Definitive hysterectomy was suggested if NR was evident after 6 months, PR was evident after 9 months, or disease progression occurred at any time during the treatment. For patients who refused hysterectomy, a multiple disciplinary discussion was held, and alternative treatments were considered. Once a patient achieved CR, the same regimen was continued for another 2–3 months for consolidation. Hysteroscopy was performed three months after the first CR. If CR was confirmed, patients were told to prepare for pregnancy or assisted reproductive technology as soon as possible.

### 2.4 Maintenance and follow-up

Low-dose cyclic progestin, oral contraceptives, or the LNG-IUS, were administered to patients without a recent pregnancy plan or after delivery to prevent recurrence. The patient was followed-up every 3 to 6 months. Ultrasound and endometrial biopsy was performed with a Pipelle to allow evaluation of the endometrium. All patients were followed-up until December 2022.

### 2.5 Molecular classification

Using the NGS classification panel, patients were classified into one of four molecular subtypes: (1) *POLE* mut, (2) *MSI-H*, (3) *TP53* wt, and (4) *TP53* abn (13).

### 2.6 Gene sequencing

Genomic DNA (tumor cell content ≥30%) from paraffin-embedded (FFPE) tissue samples was extracted, purified, and quantified using an Endometrial Cancer Molecular Classification Gene Mutation Detection Kit (Xiamen SpaceGen Co., Xiamen, China). DNA sequencing was performed on the NextSeq500 Illumina platform (Illumina Trading (Shanghai) Co., Ltd., Shanghai, China). The sequencing depth was up to 5000X, with an appropriate sensitivity to identify variants with a mutation frequency as low as 1%. We detected a range of genes related to the molecular classification of EC, including *POLE*, *TP53*, *MLH1*, *MSH2*, *PMS2*, *MSH6*, *EPCAM*, *PIK3CA*, *PTEN*, and *KRAS*. The Promega MSI Analysis System (version 1.2) was deployed on Biosystems 3500 and 3500xL Genetic Analyzers (Thermo Fisher Scientific) to identify microsatellite status. This sequencing strategy screened for mutations with a frequency > 1%, and pathological (P)/likely pathological (LP)/uncertain significant (VUS) variants were defined based on the current knowledge of relevant genes and clinical data (20, 21).

### 2.7 IHC analysis

IHC staining was performed on FFPE tissue specimens using a range of monoclonal antibodies: MLH1 (DAKO-ES05), PMS2 (DAKO-EPS1), MSH2 (DAKO-FE11), MSH6 (DAKO-EP49), p53 (DAKO-DO-7), and PTEN (DAKO-6H2.1), and utilizing a Leica Bond Max detection system. We also used two additional antibodies: estrogen receptor (ER) (Novocastra, NCL-ER-6F11) and progesterone receptor (PgR) (Novocastra, NCL-L-PGR-312). To analyze MMR (mismatch repair) proteins, the nuclear positivity of MMR proteins in more than 5% of cancer cells was used as a criterion for intact expression. Normal lymphocytes and/or stromal cells were used as internal positive controls. The overexpression pattern of p53 was defined as diffuse and strong nuclear staining in more than 80% of tumor cell nuclei; when no staining was observed, then we defined a sample as having a complete absence pattern. weak focal positive staining was defined as a wild-type pattern.

### 2.8 Statistical analysis

Continuous variables are given as medians and ranges. Categorical variables are presented as frequencies and percentages. Differences in the descriptive variables between the two groups were analyzed by the Student’s t-test or the Mann–Whitney U test, and differences between than two groups were detected by one-way analysis of variance (ANOVA) or the Kruskal–Wallis H test where appropriate. Kaplan-Meier curves were used to estimate and present therapeutic durations and the differences between groups were compared by log-rank tests. The Cox regression model was used to estimate the hazard ratios (HRs) for CR. Statistical significance was considered as a P-value < 0.05 (two-tailed). All statistical analyses were performed in SPSS (version 25.0, IBM, Armonk, NY, USA).

## 3 Results

**Figure 1:**
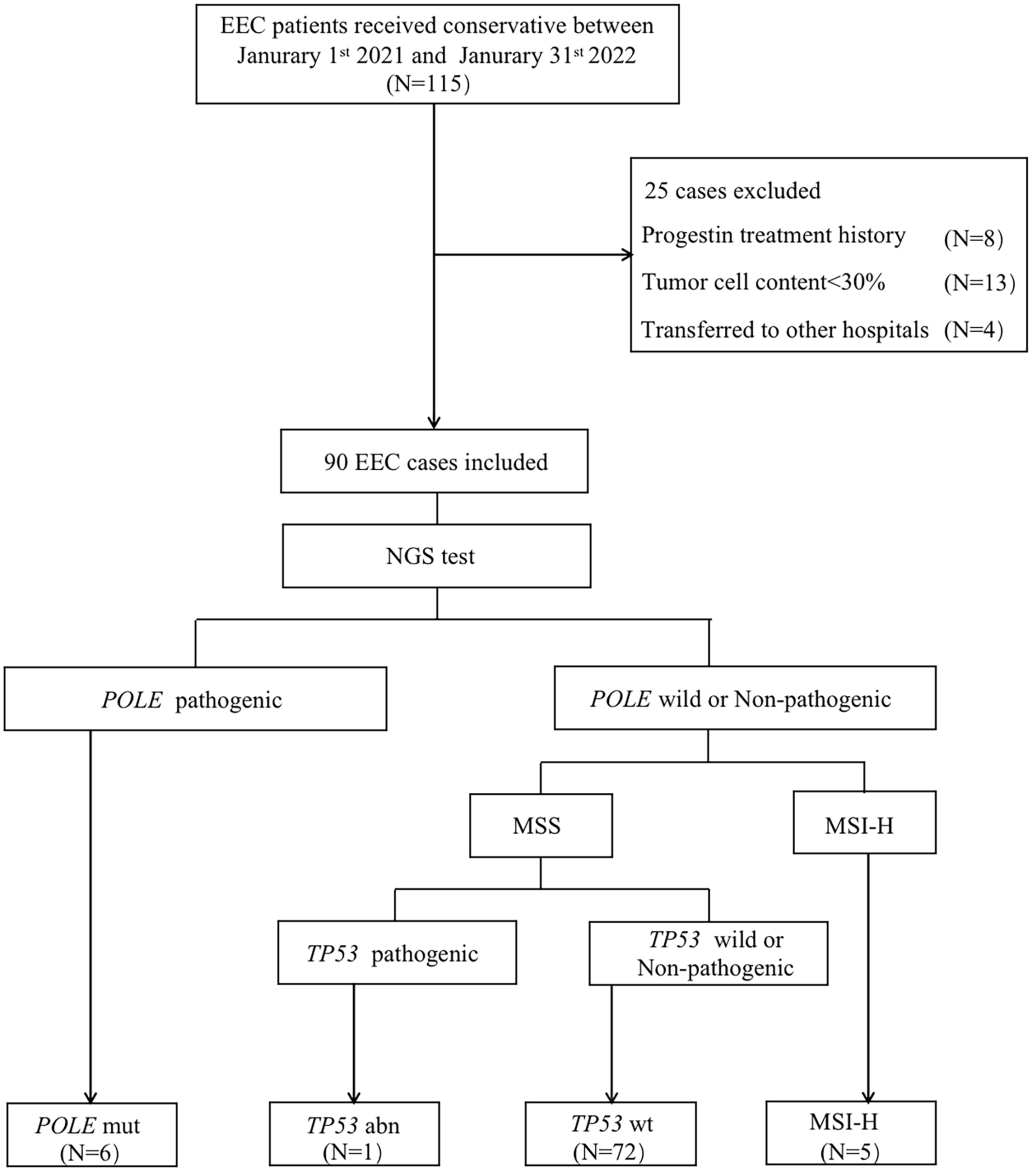
Flow chart showing the process used for patient selection. EEC, endometrioid endometrial cancer; NGS, next generation sequencing; *POLE* mut, DNA polymerase epsilon mutation; MSI-H, high microsatellite instability; MSS, microsatellite stable; *TP53* abn, *TP53* abnormal; *TP53* WT, *TP53* wildtype.

A study flow chart is presented in **Figure 1**. A total of 115 EEC patients receiving FST at the Obstetrics and Gynecology Hospital of Fudan University between January 2021 and January 2022 were retrospectively investigated. Overall, 25 cases were excluded, including eight patients who had a history of local or systemic progestin treatment for more than one month, four patients who transferred to another hospital, and 13 patients whose specimens could not be sequences or had insufficient tumor tissue for DNA extraction. Ultimately, 90 cases were included in this study. Six (6.7%) patients had *POLE* mutation, five (5.6%) patients were classified as MSI-H, 84 (86.7%) patients were classified as *TP53* wt, and one patient (1.1%) was classified as *TP53* abn.

### 3.1 Patient clinical characteristics

The characteristics of the patients are presented in **Table 1**. In our study cohort, 36 patients were over 30 years-of-age at the time of treatment. Overweight patients accounted for 47.8% of the cohort, while obese patients accounted for 13.3% of the cohort. In total, 40.0% of patients had insulin resistance, and 60.0% had hyperlipidemia. All patients had positive estrogen and progesterone receptor expression prior to the first administration of hormone therapy. Overall, 70.0% of patients in our study cohort received MA as the main FST, while another 20.0% received a combination therapy of GnRH-a and letrozole. When considering the four subgroups, a significant difference was observed for the initial treatment regimens, with the highest proportion of patients in the *TP53* wt group receiving MA or GnRH-a combined with letrozole as the initial treatment (P=0.005). After 16 weeks of treatment, the *TP53* wt group featured 32.0% of patients achieving CR, although no statistically significant difference was observed compared to other subgroups. After 32 weeks of treatment, the CR rates for the four subgroups were as follows: *POLE* mut vs. MSI-H vs. *TP53* wt vs. *TP53* abn: 50.0% (3/6) vs. 60.0% (3/5) vs. 57.7% (45/78) vs. 0 (P=0.881). During follow-up, one patient in the *TP53* abn group did not achieve CR; in contrast, the rates of CR in the other three subgroups all exceeded 80.0% (P=0.035). A total of 12 patients experienced disease recurrence after achieving CR, with a recurrence rate of 60.0% observed in the MSI-H group; this was significantly higher than that in the *POLE* mut and *TP53* wt subgroups (P=0.037). During the treatment process, disease progression occurred in 12 patients, featuring 50.0% and 40.0% of patients in the *POLE* mut and MSI-H subgroups, respectively; this compared to only 9% in the *TP53* wt subgroup (P=0.013). The median follow-up period for all patients was 59.8 weeks (range: 19.1-104.0 weeks).

**Table 1:** Patients’ clinical characteristics in fertility-preserving patients (N=90) cohort according to NGS-based molecular classification.

Variable	Total cohort N=90 (100)	*POLE* mut 6 (6.7)	MSI-H 5 (5.6)	*TP53* wt 78 (86.7)	*TP53* abn 1 (1.1)	P value
Age(years)	31 (22-42)	36.5 (26-42)	36 (30-40)	30 (22-42)	33	0.107
≥30	36 (40.0)	4 (66.7)	5 (100.0)	44 (56.4)	1 (100.0)	0.201
BMI(kg/m^2^)	24.8 (15.9-40.9)	26.3 (16.9-29.7)	26.4 (22.5-30.0)	24.52 (15.9-40.9)	35.4	0.345
25-30	43 (47.8)	4 (66.7)	2 (40.0)	25 (32.1)	0	0.200
≥30	12 (13.3)	0	1 (20.0)	10 (12.8)	1 (100.0)	
IR:N(%)	36 (40.0)	2 (33.3)	2 (40.0)	31 (39.7)	1 (100.0)	0.823
MetS:N(%)	21 (23.3)	1 (16.7)	2 (40.0)	17 (21.8)	1 (100.0)	0.247
PCOS:N(%)	18 (20.0)	0	0	18 (23.1)	0	0.458
Hyperlipidemia:N(%)	56 (62.2)	2 (33.3)	3 (60.0)	50 (64.1)	1 (100.0)	0.402
ER expression:N(%)						-
Negative	0	0	0	0	0	
Positive	90 (100.0)	6 (100.0)	5 (100.0)	78 (100.0)	1 (100.0)	
PgR expression:N(%)						-
Negative	0	0	0	0	0	
Positive	90 (100.0)	6 (100.0)	5 (100.0)	78 (100.0)	1 (100.0)	
E2(pmol/L)	154.0 (2.0-1324.0)	187.0 (12.5-1324.0)	155.0 (93.0-423.0)	153.5 (2.0-1241.0)	202.0	0.936
P(nmol/L)	1.21 (0.01-48.73)	1.3 (0.06-3.66)	0.41 (0.07-5.14)	1.18 (0.01-48.73)	2.91	0.571
T(nmol/L)	1.45 (0.01-4.86)	1.2 (0.66-2.19)	0.62 (0.03-1.95)	1.49 (0.01-4.86)	2.55	0.385
CA-125(U/ml)	18.29 (1.01-261.3)	15.81 (10.66-51.52)	13.48 (11.0-62.2)	19.22 (1.01-261.3)	47.57	0.420
HE4(pmol/L)	45.6 (24.2-281.0)	47.1 (40.5-81.7)	45.2 (27.9-55.5)	45.6 (24.7-281.0)	24.2	0.268
Therapy						**0.005**
MA	62 (68.9)	3 (50.0)	2 (40.0)	56 (71.8)	1 (100.0)	
MPA	2 (2.2)	1 (16.7)	1 (20.0)	0	0	
LNG-IUS	3 (3.3)	1 (16.7)	1 (20.0)	1 (1.3)	0	
MA+LNG-IUS	4 (4.4)	0	1 (20.0)	3 (3.8)	0	
GnRH-a+Letrozole	19 (21.1)	1 (16.7)	0	18 (23.1)	0	
CR rate						
16w	25 (27.8)	0	0	25 (32.1)	0	0.173
32w	51 (56.7)	3 (50.0)	3 (60.0)	45 (57.7)	0	0.881
Therapy outcomes						
Overall outcomes						** **
CR	85 (94.4)	5 (83.3)	5 (100.0)	75 (96.2)	0	**0.035**
Recurrence	12 (14.1)	0	3 (60.0)	9 (12.0)	/	**0.037**
Progression	12 (13.3)	3 (50.0)	2 (40.0)	7 (9.0)	0	**0.013**
Oncological outcomes at 16weeks						0.123
CR	25 (27.8)	0	0	25 (32.1)	0	** **
NR	28 (31.1)	3 (50.0)	3 (60.0)	21 (26.9)	1 (100.0)	** **
PR	34 (37.8)	2 (33.3)	2 (40.0)	30 (38.5)	0	** **
PD	3 (3.3)	1 (16.7)	0	2 (2.7)	0	** **
Oncological outcomes at 32weeks						0.325
CR	56 (62.2)	4 (66.7)	3 (60.0)	49 (62.8)	0	** **
NR	9 (10.0)	2 (33.3)	0	7 (9.0)	0	
PR	22 (24.4)	0	2 (40.0)	19 (24.4)	1 (100.0)	
PD	3 (3.3)	0	0	3 (3.8)	0	
Follow-up period(weeks)	59.8 (19.1-104.0)	39.4 (26.7-84.3)	65.7 (49.1-100.4)	59.9 (19.1-104.0)	93	0.131

Values are presented as median (range) or number (%). P-value among different groups was calculated by one-way ANOVA, Kruskal-Wallis, Chi-square, or Fisher’s exact test.

BMI, body mass index; IR, insulin resistance; MetS, metabolic syndrome; PCOS, polycystic ovary syndrome; ER, estrogen receptor; PgR, progesterone receptor; E2, Estradiol; P, Progesterone; T, Testosterone; MA, megestrol acetate; MPA, medroxyprogesterone acetate; LNG-IUS, levonorgestrel intrauterine system; GnRH-a, Gonadotropin-releasing hormone analogues; CR, complete response; NR, no response; PR, partial response; PD, progressive disease; *POLE* mut, DNA polymerase epsilon mutation; MSI-H, high microsatellite instability; *TP53* wt, *TP53* wildtype; *TP53* abn, *TP53* abnormal.

### 3.2 Molecular and tppathological characteristics

**Figure 2:**
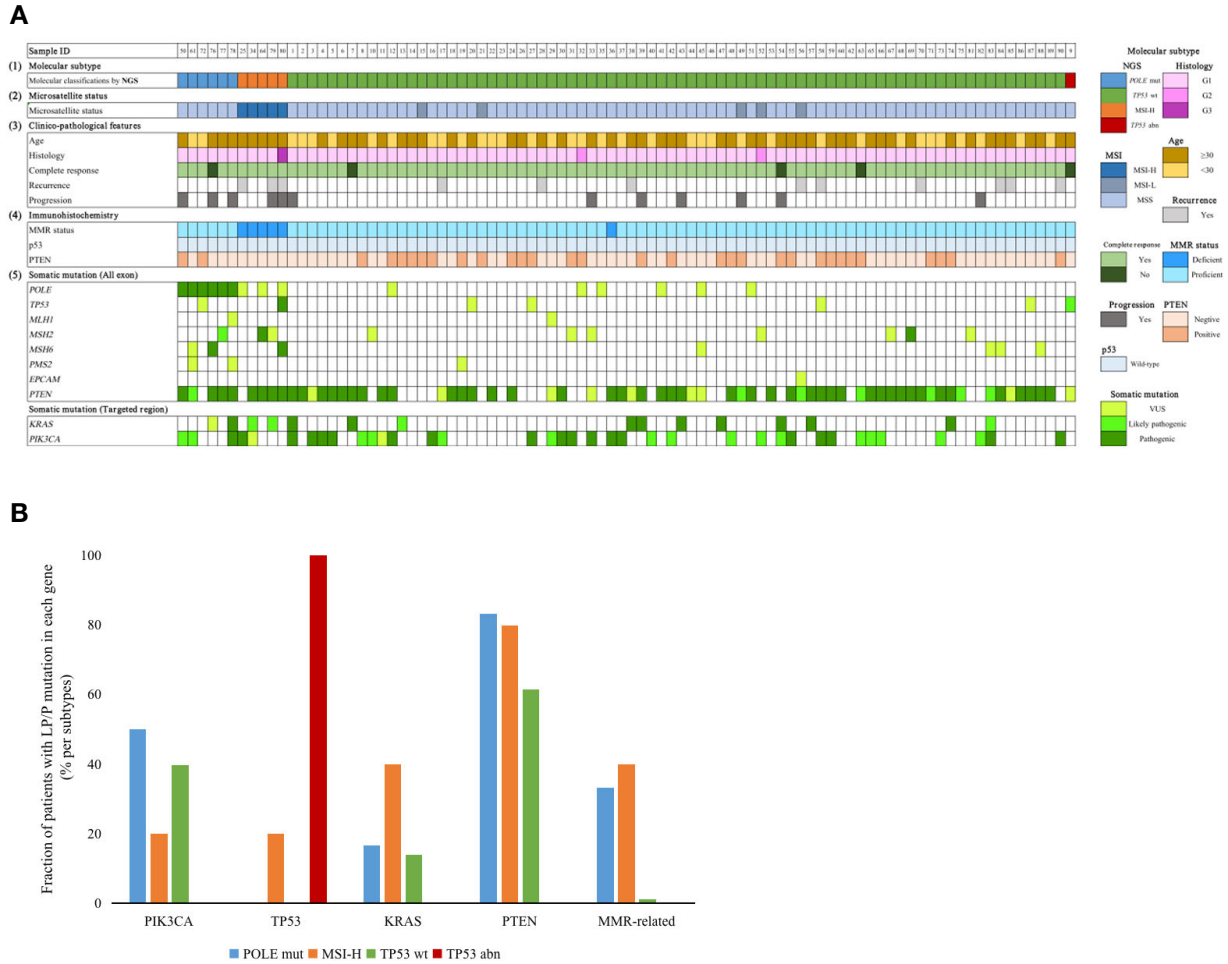
**(A)** Clinicopathological factors and mutation profiles in our cohort. (1) Molecular subtype by NGS. (2) Microsatellite status. (3) Clinical factors, CR status, recurrence status and progression status. (4) IHC staining. (5) Mutation profiles of the 90 EEC patients. **(B)** Distribution of different genes with P/LP mutations in the four subgroups. NGS, next generation sequencing; MMR, mismatch repair; *POLE* mut, DNA polymerase epsilon mutation; MSI-H, high microsatellite instability; MSI-L, low microsatellite instability; MSS, microsatellite stable; *TP53* abn, *TP53* abnormal; *TP53* WT, *TP53* wildtype.

The somatic mutation results for all patients in the study cohort are shown in **Figure 2**. Patients in the MSI-H group were all 30 years-of-age or older. One patient in the MSI-H group experienced recurrence and progression during FST, with the pathological type progressing to grade 3. Two patients were grade 2, both were TP53 wt. During follow-up, a total of five patients failed to achieve CR, one was classified as *POLE* mut, three were classified as *TP53* wt, and one was classified as *TP53* abn. In terms of IHC results, five patients showed a loss of MMR-related protein. All patients exhibited wild-type p53 expression.

When considering our target genes, *PTEN* was the gene with the highest frequency of P/LP mutations; this was detected in 57/90 (63.3%) patients; this was followed by *PIK3CA*, *KRAS*, *POLE*, *MMR*-related, and *TP53*, detected in 35/90 (38.9%), 14/90 (15.6%), 6/90 (6.7%), 5/90 (5.6%) and 2/90 (2.2%) patients, respectively. Fourteen patients did not have any P/LP mutations according to our gene panel. *PIK3CA* and *PTEN* mutations were more frequent in the *POLE* mut group; however, this was not statistically significant. It appeared that the presence of *TP53* P/LP mutations was an unfavorable factor for the outcome of FST. One MSI-H patient, with a concurrent *TP53* pathogenic mutation, experienced disease recurrence and progression after achieving CR, while another patient classified as *TP53*abn did not achieve CR during the follow-up period.

### 3.3 The effects of related factors on treatment outcomes

**Table 2** shows the associations between variables and FST outcomes. LP/P *PTEN* mutations significantly reduced the CR rate at 16 weeks of treatment (P=0.002). The CR rate at 32 weeks of treatment decreased significantly with increasing BMI (P=0.004) and insulin resistance (P=0.005). Surprisingly, the combination of GnRH-a and letrozole as the initial treatment resulted in a 100.0% CR rate at 32 weeks (P<0.001); this was significantly higher than any of the other therapies. In our cohort, all patients who progressed had pathological progression, but no evidence of metastasis was found by enhanced MRI. There was a significant difference in disease progression rates among different initial therapies (P=0.011), with lower rates observed in the MA and GnRH-a combined with letrozole groups. At the molecular level, LP/P mutations in *POLE*, *KRAS*, and *MMR-*related genes were significantly associated with disease progression (P=0.013, P=0.002, P=0.029, respectively). In addition, MSI-H patients had a higher recurrence rate after CR (60.0%, P=0.005). There were significant differences in recurrence rates among different initial treatment and maintenance therapies; patients who were treated with MA as the initial treatment and Diane-35 plus metformin as the maintenance treatment after CR had a significantly lower recurrence rate (P=0.005; P=0.008, respectively).

**Table 2:** Factors associated with fertility-sparing treatment outcomes.

Variables			16-week CR rate	P value	32-week CR rate	P value	Progression	P value	Recurrence	P value
Age≥30		YES	22.2% (12/54)	0.150	53.7% (29/54)	0.487	16.7% (9/54)	0.411	18.0% (9/50)	0.362
		NO	36.1% (13/36)		61.1% (22/36)		8.3% (3/36)		8.6% (3/35)	
BMI		<25	38.3% (18/47)	0.066	72.3% (34/47)	**0.004**	10.6% (5/47)	0.143	8.7% (4/46)	0.161
		25-30	16.1% (5/31)		45.2% (14/31)		22.6% (7/31)		24.1% (7/29)	
		≥30	16.7% (2/12)		25.0% (3/12)		0		10.0% (1/10)	
IR		YES	16.7% (6/36)	0.055	38.9% (14/36)	**0.005**	16.7% (6/36)	0.532	12.5% (4/32)	0.991
		NO	35.2% (19/54)		68.5% (37/54)		11.1% (6/54)		15.1% (8/53)	
MetS		YES	23.8% (5/21)	0.643	42.9% (9/21)	0.145	19.0% (4/21)	0.608	21.1% (4/19)	0.541
		NO	29.0% (20/69)		60.9% (42/69)		11.6% (8/69)		12.1% (8/66)	
PCOS		YES	33.3% (6/18)	0.556	50.0% (9/18)	0.523	5.6% (1/18)	0.485	22.2% (4/18)	0.465
		NO	26.4% (19/72)		58.3 (42/72)		15.3% (11/72)		11.9% (8/67)	
Hyperlipidemia		YES	28.6% (16/56)	0.829	57.1% (32/56)	0.907	14.3% (8/56)	0.983	15.1% (8/53)	0.991
		NO	26.5% (9/34)		55.9% (19/34)		11.8% (4/34)		12.5% (4/32)	
Therapy		MA	21.0% (13/62)	0.073	43.5% (27/62)	<0.001	12.9% (8/62)	**0.011**	8.6% (5/58)	**0.005**
		MPA	0		50.0% (1/2)		50.0% (1/2)		0	
		LNG-IUS	33.3% (1/3)		66.7% (1/3)		66.7% (2/3)		100.0% (2/2)	
		MA+LNG-IUS	25.0% (1/4)		50.0% (2/4)		25.0% (1/4)		50.0% (2/4)	
		GnRH-a+Letrozole	52.6% (10/19)		100.0% (19/19)		0		15.8% (3/19)	
Molecular classification		POLE	0	0.173	50.0% (3/6)	0.881	50.0% (3/6)	**0.013**	0	**0.037**
		MSI-H	0		60.0% (3/5)		40.0% (2/5)		60.0% (3/5)	
		TP53 wt	32.1% (25/78)		57.7% (45/78)		9.0% (7/78)		12.0% (9/75)	
		TP53 abn	0		0		0			
LP/P somatic mutant		YES	23.7% (18/76)	0.090	52.6% (40/76)	0.072	15.8% (12/76)	0.242	14.1% (10/71)	1.000
* *		NO	50.0% (7/14)		78.6% (11/14)		0		14.3% (2/14)	
*PTEN*		YES	16.4% (9/55)	**0.002**	49.1% (27/55)	0.069	16.4% (9/55)	0.458	11.5% (6/52)	0.591
		NO	45.7% (16/35)		68.6% (24/35)		8.6% (3/35)		18.2% (6/33)	
*PIK3CA*		YES	22.9% (8/35)	0.406	48.6% (17/35)	0.216	17.1% (6/35)	0.596	12.1% (4/33)	0.919
		NO	30.9% (17/55)		61.8% (34/55)		10.9% (6/55)		15.4% (8/52)	
*KRAS*		YES	21.4% (3/14)	0.801	35.7% (5/14)	0.085	42.9% (6/14)	**0.002**	16.7% (2/12)	1.000
		NO	28.9% (22/76)		60.5% (46/76)		7.9% (6/76)		13.7% (10/73)	
MMR-related		YES	0	0.271	66.7% (4/6)	0.932	50.0% (3/6)	**0.029**	20.0% (1/5)	0.542
		NO	29.8% (25/84)		56.0% (47/84)		10.7% (9/84)		13.8% (11/80)	
*TP53*		YES	0	1.000	50.0% (1/2)	1.000	50.0% (1/2)	0.250	100.0% (1/1)	0.141
		NO	28.4% (25/88)		56.8% (50/88)		12.5% (11/88)		13.1% (11/84)	
Maintenance Therapy		Dydrogesterone	–		–		–		20.0% (1/5)	**0.002**
		LNG-IUS							27.8% (5/18)	
		Diane-35 + metformin							6.7% (4/60)	
		Diane-35 + metformin + LNG-IUS							100.0% (1/1)	
		none							100.0% (1/1)	

BMI, body mass index; IR, insulin resistance; MetS, metabolic syndrome; PCOS, polycystic ovary syndrome; MA, megestrol acetate; MPA, medroxyprogesterone acetate; LNG-IUS, levonorgestrel intrauterine system; GnRH-a, Gonadotropin-releasing hormone analogues; LP, likely pathogenic; P, pathogenic; MMR, mismatch repair.

Bold values means the P-value < 0.05, there is a statistical difference.

**Figure 3:**
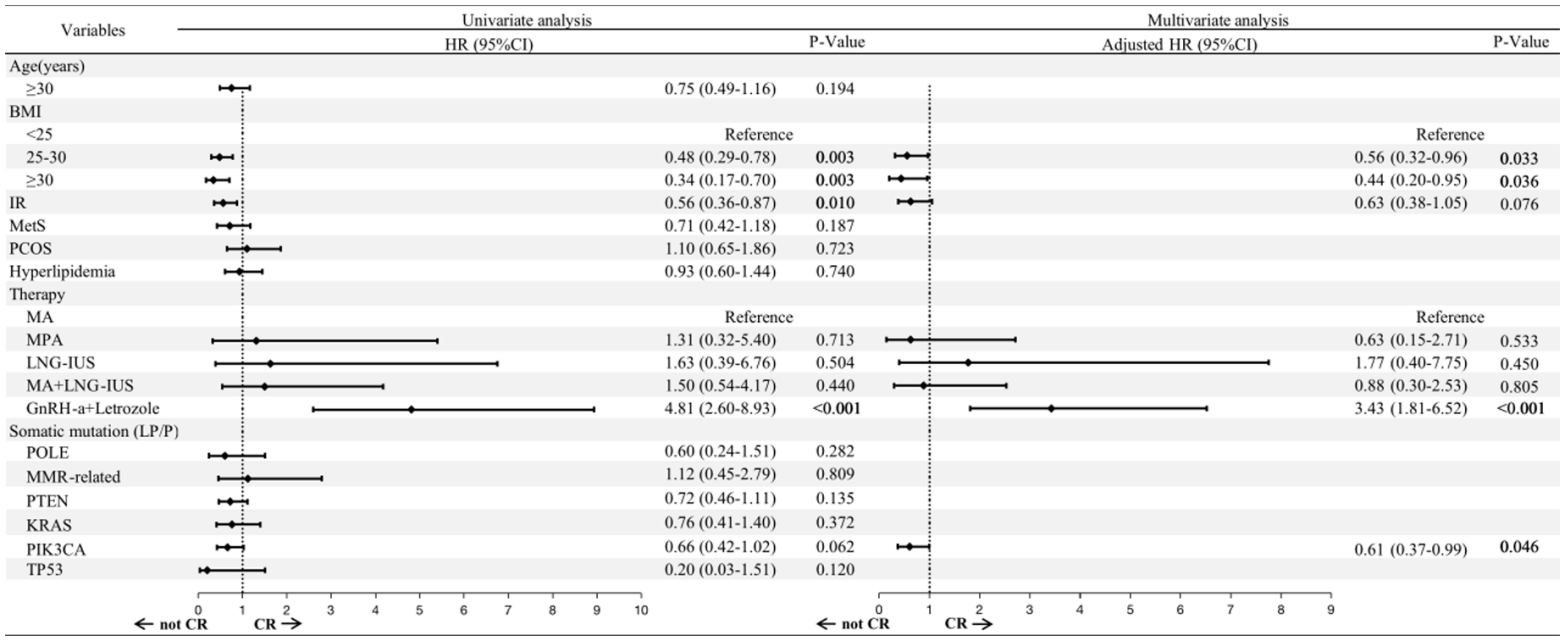
Risk factors associated with the FST outcomes, as determined by Cox regression. BMI, body mass index; IR, insulin resistance; MetS, metabolic syndrome; PCOS, polycystic ovary syndrome; MA, megestrol acetate; MPA, medroxyprogesterone acetate; LNG-IUS, levonorgestrel intrauterine system; GnRH-a, Gonadotropin-releasing hormone analogues; LP, likely pathogenic; P, pathogenic; HR, hazard ratio; CI, confidence interval.

Univariate Cox regression analysis demonstrated that overweight (HR: 0.48; 95% CI: 0.29-0.78; P=0.003), obesity (HR: 0.34; 95% CI: 0.17-0.70; P=0.003) and IR (HR: 0.56; 95% CI: 0.36-0.87; P=0.010) were associated with a lower cumulative CR rate. While GnRH-a combined with letrozole was significantly associated with a higher cumulative CR rate (HR: 4.81; 95% CI: 2.60-8.93; P<0.001) (**Figure 3**).

We selected factors with P<0.01 in the univariate Cox regression for further multivariate analysis. The adverse effects of overweight (HR: 0.56; 95% CI: 0.32-0.96; P=0.033) and obesity (HR: 0.44; 95% CI: 0.20-0.95; P =0.036) remained significant. It is worth noting that *PIK3CA* mutation was associated with a lower cumulative CR rate after multivariate Cox regression (HR: 0.61; 95% CI: 0.37-0.99; P =0.046). GnRH-a combined with letrozole was still considered a favorable factor for CR (HR: 3.43; 95% CI: 1.81-6.52; P<0.001).

### 3.4 The effects of different molecular mutants on the outcomes of oncological treatment

**Figure 4:**
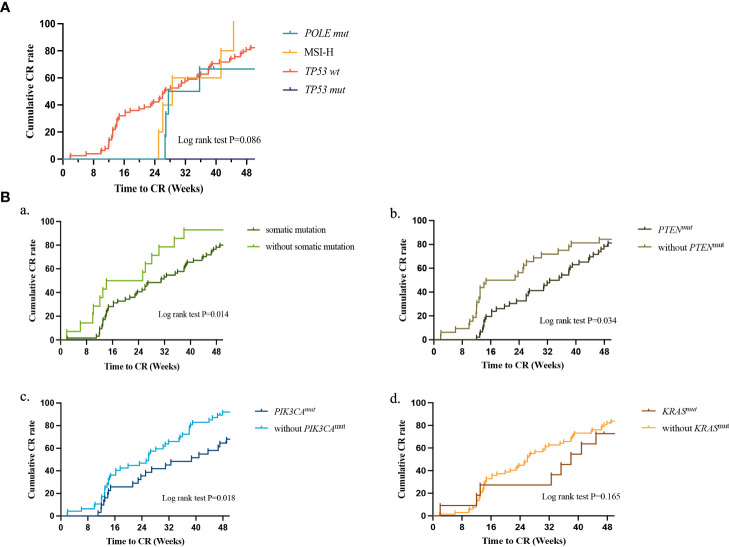
**(A)** Treatment duration to CR between different molecular classifications as determined by Kaplan-Meier analysis and compared by the log-rank test. *POLE* mut, DNA polymerase epsilon mutation; MSI-H, high microsatellite instability; *TP53* abn, *TP53* abnormal; *TP53* WT, *TP53* wildtype; CR, complete response. **(B)** Treatment duration to CR between different somatic mutants as determined by Kaplan-Meier analysis and compared by the log-rank test in *TP53* WT. (a) patients with or without somatic mutation; (b) patients with or without *PTEN* mutation; (c) patients with or without *PIK3CA* mutation; (d) patients with or without *KRAS* mutation. CR, complete response.

The treatment durations of the four molecular subtypes are presented in **Figure 4A**. Following multivariate Cox regression analysis, we found that the initial treatment significantly influenced the CR rate. Thus, our analysis excluded patients who received GnRH-a combined with letrozole as the initial therapy. No significant difference was found in the time to achieve CR when compared across the four subtypes (log rank test; P=0.086); however, we did find that only patients in the *TP53* wt group achieved CR at 24 weeks of treatment. Further analysis of the molecular characteristics of the *TP53* wt group (**Figure 4B**) showed that patients without targeted gene mutations had a shorter duration of treatment (log rank P=0.014), while *PTEN* and *PIK3CA* mutations prolonged the duration of treatment when conservative therapy was administered (log rank test: P=0.034; log rank test: P=0.018).

### 3.5 Patients with *POLE* mut, MSI-H, and *TP53* abn

**Supplementary Table 1** presents the treatment details of patients with *POLE* mut, MSI-H, and *TP53* abn in our study. In our cohort, the P286R mutation accounted for 66.6% of all *POLE* mut cases, with M444K and V411L mutations in the remaining two cases. All five MSI-H patients underwent germline genetic testing, and one patient was diagnosed with Lynch syndrome (LS). Three *POLE* mut and two MSI-H patients changed therapy during treatment. One *POLE* mut patient received hysterectomy due to a failure to respond after nine months of treatment, while another LS patient underwent surgery due to disease recurrence and progression. Histopathological examination showed no deep myometrial invasion, lymphovascular space invasion (LVSI), or metastasis to the ovaries or lymph nodes.

### 3.6 Details of patients who underwent a change in therapeutic regimen

Next, we analyzed patients who changed their therapeutic regimen during treatment following multidisciplinary discussion. A total of 19 patients changed their therapy, with three (50%) in the *POLE* mut subgroup, two (40%) in the MSI-H subgroup, and 14 (17.9%) in the *TP53* wt subgroup, as shown in **Supplementary Figure 1**. At the final follow-up, 89.5% of patients achieved CR following a change in therapy. Two patients each in the *POLE* mut and *TP53* WT subgroups changed their treatment due to disease progression. Sixteen patients switched to GnRH-a combined with letrozole, while three patients switched to a combination therapy featuring Diane-35 and metformin. We noticed that two patients still did not achieve CR within the follow-up period after changing therapeutic regime.

## 4 Discussion

### 4.1 Molecular characteristics and the outcomes of conservative treatment

In this study, we demonstrated that molecular classification can be used to predict the prognoses of patients with EC when treated conservatively. Our findings are different from those of previous studies in that patients with *POLE* mutation did not show a better response to progesterone therapy and were instead found to be insensitive to such treatment. Kaplan-Meier survival curve analysis did not reveal a significant difference among the four subgroups; this may have been related to a change of treatment and the small size of the patient cohort. In our study cohort, only one patient in the *POLE* mut group benefited from high-dose progesterone therapy. Furthermore, in the *POLE* mut group, patients with AEH were more likely to progress to EC during treatment; this was in stark contrast to the patients undergoing surgery. By analyzing existing reports, we only found three cases of conservatively treated EC patients with *POLE* mutations, two of whom received oral progesterone therapy but eventually underwent surgical treatment due to disease recurrence or progression; furthermore, only one patient achieved CR without recurrence after six months of treatment with LNG-IUS (16, 22). These data suggested that *POLE* mutation may be one of the unfavorable factors of FST in EC patients. Similar to surgical patients, those with *TP53* mutation were found to be associated with a poor outcome. One patient with EC was classified as a *TP53* abn in our study and did not achieve CR even after 90 weeks of treatment; this patient did not change her therapeutic regime due to irregular follow-up. Another patient with MSI-H and *TP53* mutation experienced disease recurrence and progression to high-grade EEC during treatment. In addition, we found that 60.0% (3/5) of patients in the MSI-H group experienced disease recurrence; this finding was consistent with previously reported research findings (17).

We found that patients in the *POLE* mut and MSI-H subgroups had a high tumor mutation burden (TMB), with the former showing a higher burden. In a recent study, Riggs et al. reported that the high TMB was associated with an increased frequency of *DACH1* gene mutation (23). The *DACH1* gene was positively correlated with progesterone receptor expression (24), thus suggesting that the insensitivity of patients in the *POLE* mut and MSI-H subgroups to progesterone may be related to mutation of the *DACH1* gene. Moreover, the detection of *DACH1* gene mutations can facilitate the identification of appropriate patients for FST. The lower TMB in the MSI-H subgroup when compared to the *POLE* mut subgroup may be correlated with the difference in long-term treatment efficacy between the two groups. Recently, Hu et al. reported that the expression of *PDGFC*, *DIO2*, *SOX9*, and *BCL11A* was upregulated in progesterone-insensitive endometrial lesions when compared with progesterone-sensitive endometrial lesions, while the expression of *FOXO1*, *IRS2*, *APOE*, *FYN*, and *KLF4* was downregulated, as based on the integrated analysis of ATAC-Seq and RNA-Seq (25). By conducting differential gene enrichment analysis of TCGA data (the analytical data were not presented in this article), we found that the expression of *IRS2* was downregulated in *POLE* mut and MSI-H groups of EC patients compared with the *TP53* wt group. *IRS2* acts downstream of insulin receptor, activating the MAPK and PI3K/AKT pathways and inducing glucose uptake and membrane marker expression (26). Our study suggested that the downregulation of *IRS2* may be one of the reasons for the poor response to progesterone conservation therapy in the *POLE* mut and MSI-H groups.

Further analysis based on NGS results showed that patients with likely pathogenic/pathogenic gene mutations had longer treatment duration to achieve CR compared to those without any mutations, and patients with *PTEN* mutation had significantly lower response rates compared to those without *PTEN* mutation, consistent with our previous study (27). Additionally, our study found that *PIK3CA* mutation is also one of the factors that prolong the treatment duration.

In addition, AEH, a precursor to endometrial cancer, was found concurrent EC in approximately 32.6% of patients (28). However, studies on the molecular characterization of these patients are still limited. In a retrospective analysis conducted by Puechl AM and colleagues, of 37 patients with AEH, one of two (50%) patients with MMR-D demonstrated disease progression, one of four (25%) patients with POLE mutations experienced disease progression, and only two of 27 (7.4%) patients with p53wt demonstrated disease progression (29). Our study confirms these findings, suggesting that AEH patients with POLE mutations and MSI-H are more likely to experience disease progression. However, due to the limited sample size, further studies are essential to explore this patient subgroup in depth, potentially for providing valuable prognostic insights and facilitating the development of more personalized treatment and follow-up strategies based on molecular features.

### 4.2 Molecular characteristics and disease prognoses

For patients undergoing surgery, different molecular subtypes were associated with different prognoses, the *POLE* mut group was associated with a higher 10-year recurrence-free survival (RFS) rate, whereas the p53mut EC patients presented with a higher rate of distant recurrence and lower overall survival (30). In our study, LP/P mutations in *POLE*, *KRAS*, and MMR-related genes were associated with higher rates of disease progression. Following Cox multivariate regression analysis, *PIK3CA* was identified as a risk factor for achieving CR in endometrial cancer. Of the four subtypes, the *TP53* wt group had the largest number of patients. A previous study showed that patients with mutant *KRAS* and wild-type *ARID1A* were associated with a poorer 5-year recurrence-free survival in a *TP53* wt group (31). And our study identified *PTEN* and *PIK3CA* mutations as new molecular markers affecting the outcome of FST in patients with EC. *PTEN*, a tumor suppressor gene, was reported to be mutated in 57–83% of all cases of EC and is the most common molecular event in early endometrial cancer (32). *PTEN* is also a known negative regulator of the PI3K/AKT/mTOR pathway, and studies have reported a significant association between the loss of *PTEN* expression and metastatic disease (33). *PIK3CA* is also associated with disease invasion. For example, Hayes et al. proposed that EC with *PIK3CA* mutation should be considered as having invasive cancer, whereas those without this gene mutation would be candidates for a more conservative approach (34). In our present study, we found a correlation between *PIK3CA* mutation and poorer outcomes following conservative therapy. It has been reported that *KRAS* plays an early role in the progression of EC (30); similarly, our study cohort showed a higher rate of *KRAS* pathogenic mutations in the disease progression group.

Some patients with EC exhibited multiple molecular characteristics; previous studies showed patients with MMR-D and *POLE* mut carrying *TP53* mutations had better prognoses than single *TP53* mutation in surgical patients (35). In our study, one MSI-H patient who carried a pathogenic *TP53* mutation experienced disease progression during treatment; in this case, the histological type progressed to high-grade endometrioid carcinoma. This suggested that closer monitoring should be conducted for patients carrying *TP53* mutation. Currently, the outcomes of conservative treatment for patients with multiple molecular characteristics remain unclear and more clinical data need to be acquired and analyzed.

### 4.3 Other possible factors affecting the efficacy of conservative therapy

Our analyses confirmed the correlation between weight and FST outcome in patients with EC. Overweight and obesity were identified as independent risk factors that affect the duration of treatment in patients undergoing conservative treatment. Previous studies demonstrated a significant correlation between different BMI categories and the outcomes of conservative treatment (7). In our study cohort, the overweight populations in the *POLE* mut and MSI-H groups were higher than that in the *TP53* wt group, although no statistically significant difference was detected. The molecular classification of EC will allow us to focus on the correlation between different molecular characteristics and the outcomes of conservative treatment, and also considered the joint effects of molecular characteristics and metabolism on the outcomes of conservative treatment in patients with EC. The interaction between molecular characteristics and metabolism represents a significant research direction in the future.

### 4.4 The relationship between treatments and the outcomes of conservative therapy

GnRH-a, a gonadotropin-releasing hormone agonist, can suppress the pituitary secretion of gonadotropins, reduce the secretion of ovarian hormones, inhibit ovarian function, and reduce the circulating levels of estrogen. In recent years, GnRH-a has been reported as an effective FST for patients with endometrial cancer (36, 37). Our analysis demonstrated that in the *POLE* mut subgroup, the use of GnRH-a combined with letrozole as the initial treatment method yielded significantly greater benefits than progesterone. This indirectly confirmed the insensitivity of *POLE* mut to progesterone.

Our study analyzed the prognostic value of molecular classification and other molecular features for patients with EC receiving FST. However, this study also had certain limitations that need to be considered. First, our analysis was limited by its retrospective nature and the use of a single institution database; this may have induced possible bias in the selection of patients. However, detailed data recording and strict adherence to inclusion and exclusion criteria for every AEH or EEC patient were applied throughout the study to avoid selection bias. Therefore, further prospective studies are now required to validate the full impact of molecular classification for patients with EC undergoing FST.

In conclusion, our study demonstrated that the molecular classification of EC represents a useful classification system applicable to patients receiving conservative treatment. However, its guidance for prognoses should be distinguished from that of surgical patients. In addition, we found that somatic pathogenic mutations of some other genes were also associated with the prognoses of conservative treatment, including *PTEN*, *KRAS*, and *PIK3CA*. The findings of our study indicated that molecular classification has the potential to differentiate EC patients with similar histological features but different prognoses, consequently providing direction for personalized therapeutic and monitoring regimens for patients with unique molecular profiles.

## Data availability statement

The raw data supporting the conclusions of this article will be made available by the authors, without undue reservation.

## Ethics statement

The studies involving humans were approved by Ethics Committees of Obstetrics and Gynecology Hospital of Fudan University. The studies were conducted in accordance with the local legislation and institutional requirements. The participants provided their written informed consent to participate in this study.

## Author contributions

YXu: Writing – original draft, Conceptualization, Data curation, Formal Analysis, Investigation, Methodology, Project administration, Writing – review & editing. MZ: Data curation, Formal Analysis, Investigation, Project administration, Writing – original draft, Writing – review & editing, Software. LZ: Data curation, Investigation, Writing – review & editing. BW: Writing – original draft. TW: Data curation, Formal Analysis, Writing – review & editing. YXue: Data curation, Supervision, Validation, Writing – review & editing. ZX: Formal Analysis, Software, Supervision, Writing – review & editing. WS: Methodology, Project administration, Writing – review & editing. XC: Conceptualization, Project administration, Resources, Supervision, Validation, Visualization, Writing – review & editing. CW: Conceptualization, Funding acquisition, Project administration, Resources, Validation, Visualization, Writing – review & editing.

## Supplementary material

Supplementary Figure 1 Patients changed therapy in the study cohort. *POLE* mut, DNA polymerase epsilon mutation; MSI-H, high microsatellite instability; *TP53* wt, *TP53* wildtype; CR, complete response; PD, progressive disease; NR, No response; PR, partial response; GnRH-a, Gonadotropin-releasing hormone analogues.
