# Supplementary figures and images for: Outcomes of fertility preservation treatments in patients with endometrial cancer with different molecular classifications based on an NGS panel
Source: Front Oncol. 2023 Nov 9;13:1282356. doi: 10.3389/fonc.2023.1282356 (PMC10665890; doi:10.3389/fonc.2023.1282356)

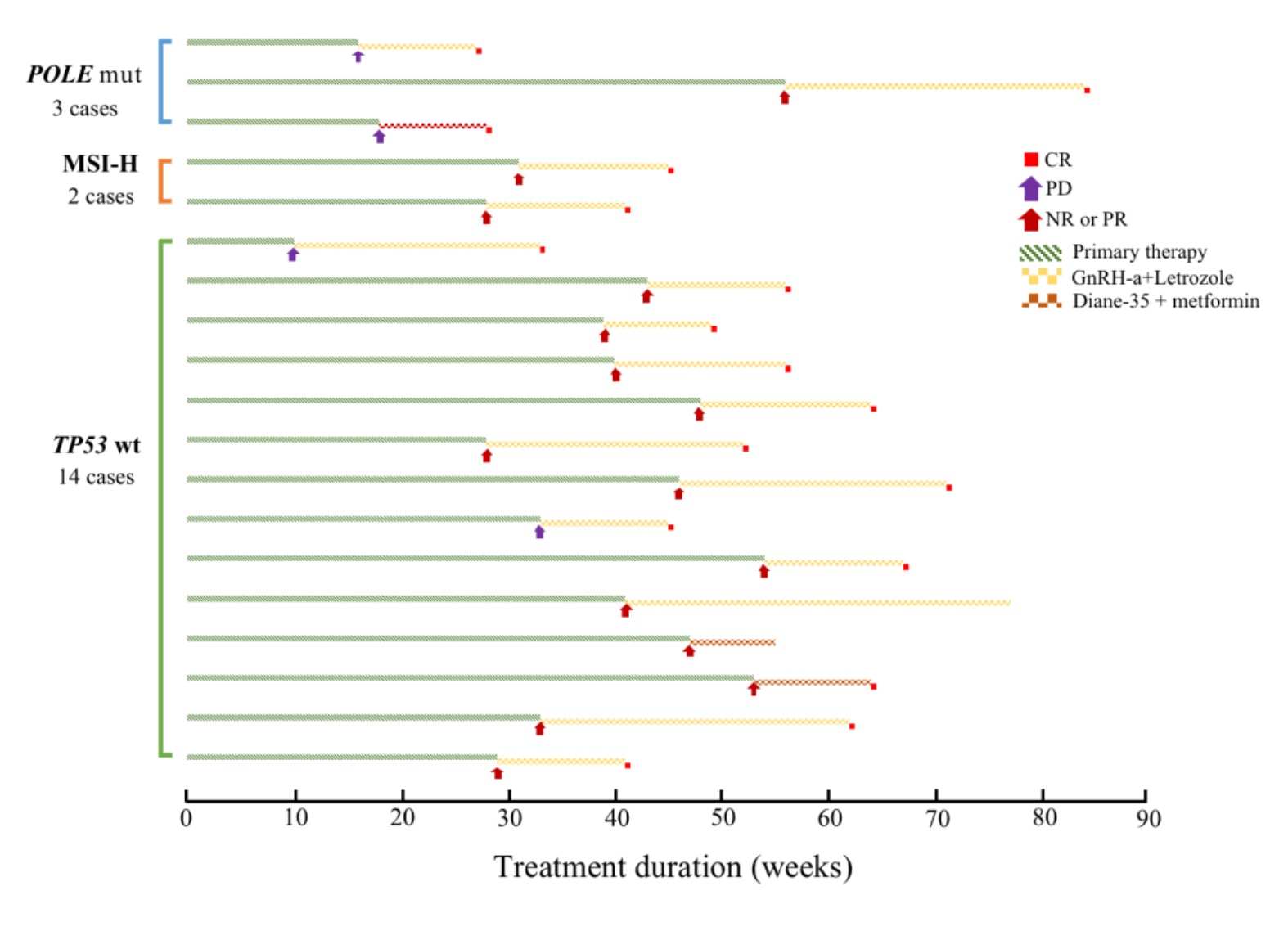

Supplement: Supplementary Figure 1 — Patients changed therapy in the study cohort. POLE mut, DNA polymerase epsilon mutation; MSI-H, high microsatellite instability; TP53 wt, TP53 wildtype; CR, complete response; PD, progressive disease; NR, No response; PR, partial response; GnRH-a, Gonadotropin-releasing hormone analogues. [file Image_1.tif]
